# Nanomechanics and Sodium Permeability of Endothelial Surface Layer Modulated by Hawthorn Extract WS 1442

**DOI:** 10.1371/journal.pone.0029972

**Published:** 2012-01-10

**Authors:** Wladimir Peters, Verena Drueppel, Kristina Kusche-Vihrog, Carola Schubert, Hans Oberleithner

**Affiliations:** 1 Institute of Physiology II, University of Münster, Münster, Germany; 2 Institute of Gender in Medicine, Charité - Universitätsmedizin Berlin, Berlin, Germany; Clarkson University, United States of America

## Abstract

The endothelial glycocalyx (eGC) plays a pivotal role in the physiology of the vasculature. By binding plasma proteins, the eGC forms the endothelial surface layer (ESL) which acts as an interface between bloodstream and endothelial cell surface. The functions of the eGC include mechanosensing of blood flow induced shear stress and thus flow dependent vasodilation. There are indications that levels of plasma sodium concentrations in the upper range of normal and beyond impair flow dependent regulation of blood pressure and may therefore increase the risk for hypertension. Substances, therefore, that prevent sodium induced endothelial dysfunction may be attractive for the treatment of cardiovascular disease. By means of combined atomic force - epifluorescence microscopy we studied the impact of the hawthorn (*Crataegus* spp.) extract WS 1442, a herbal therapeutic with unknown mechanism of action, on the mechanics of the ESL of *ex vivo* murine aortae. Furthermore, we measured the impact of WS 1442 on the sodium permeability of endothelial EA.hy 926 cell monolayer. The data show that (i) the ESL contributes by about 11% to the total endothelial barrier resistance for sodium and (ii) WS 1442 strengthens the ESL resistance for sodium up to about 45%. This mechanism may explain some of the vasoprotective actions of this herbal therapeutic.

## Introduction

The daily sodium intake in industrial countries greatly exceeds physiological needs. There is a strong correlation between the daily sodium intake and the prevalence of arterial hypertension, one of the main risk factors of cardiovascular disease and mortality worldwide [1]. One important mechanism of blood pressure regulation is blood flow dependent vasodilation, which in turn is sensitive to changes in the plasma concentration of sodium [2]. In previous studies we showed that high plasma sodium may promote aldosterone-mediated hypertension by diminishing the release of the vasodilator nitric oxide (NO) [3]. Blood flow dependent vasodilation is also controlled by heparan sulfate residues functioning as mechanosensors in the endothelial glycocalyx (eGC) [4]–[6]. The eGC is an anionic biopolymer that covers the luminal side of blood vessels. It consists of proteoglycans and glycoproteins with attached polysaccharide side chains, also referred to as glycosaminoglycans. By binding plasma constituents eGC forms the endothelial surface layer (ESL) – an interface between blood and endothelial cell surface [7]. The ESL plays a pivotal role in the endothelial barrier function including the regulation of vascular permeability [8] and extravasation of colloids and fluids [9], [10]. Furthermore, the ESL forms a regulatory microdomain for many processes on the membrane surface such as lipolysis [11], inactivation of procoagulant species [12] and recruitment of leukocytes to the subendothelium [13], [14]. Recently it was shown that high plasma sodium damages the endothelial glycocalyx [15].

Fluid shear stress induces a conformational change of heparan sulfate proteoglycans of the eGC from a random coiled state to an unfolded state. The unfolded structure reveals sodium binding sites and promotes transmembrane Na^+^-influx by counter-ion migration along the polysaccharide. This process causes a vasodilatory response [2], [16]. Thus, substances that protect the ESL and increase endothelial NO-release could provide interesting therapeutical approaches to fight sodium induced hypertension and possibly other cardiovascular diseases.

Hawthorn extracts have been used in Europe for many decades to treat cardiovascular diseases. It has been shown that hawthorn extract WS 1442 induces an endothelium dependent, NO and EDRF mediated vasorelaxation [17], [18] and is cardioprotective in ischemic reperfusion injuries [19], [20]. Hawthorn extracts exhibit antioxidative properties and inhibit neutrophil elastase [21]. The efficacy and safety of WS 1442 was demonstrated in numerous clinical studies [22]–[29]. A recently published review [30] summarizes some pharmacological and clinical data of hawthorn extracts.

To test, whether WS 1442 modulates the functional properties of the ESL, we established a method using combined atomic force microscopy (AFM) - epifluorescence microscopy. This approach makes it possible to observe in real time eGC height and stiffness [31], [32], and simultaneously, examine eGC-constituents by means of immunofluorescence . Though there is considerable knowledge about the permeability of the ESL to macromolecules [33]–[35], there are no data concerning its selective ion permeability. To test whether sodium ions can freely pass the ESL, we compared the sodium permeability (*P_Na+_*) of the endothelial cell monolayer under control conditions and under conditions where the eGC was enzymatically digested. Furthermore, we measured the *P_Na+_* of WS 1442 treated cells to analyze the link between the mechanical state of the ESL and *P_Na+_*.

## Materials and Methods

### Cell culture

Human endothelial EA.hy 926 cells (kindly provided by Cora-Jean Edgell, University of North Carolina, USA) and bovine aortic endothelial GM 7373 cells (DSMZ, Braunschweig, Germany) were cultured at 37°C and 5% CO_2_. For GM 7373, minimal essential medium (Invitrogen Corp., La Jolla, CA, USA) supplemented with 20% fetal calf serum (FCS; PAA Clone, Coelbe, Germany), 1% MEM vitamins (Invitrogen), 1% MEM nonessential amino acids (Invitrogen) and 1% Pen-Strep (Invitrogen) was used. EA.hy 926 cells were cultured in Dulbecco's modified Eagle's medium (Invitrogen) supplemented with 1% Pen-Strep solution and 10% FCS.

### Preparation of *ex vivo* aortae

All animal procedures were performed in accordance with the guidelines of the Charité, Universitätsmedizin Berlin, and were specifically approved by the Landesamt für Gesundheit und Soziales (LaGeSo, Berlin, Germany) for the use of laboratory animals (G0355/09) and followed the “Principles of Laboratory Animal Care” (NIH publication No. 86-23, revised 1985), as well as the current version of German Law on the Protection of Animals.

Mouse aortae were isolated and freed from surrounding tissue. A small patch (about 1 mm^2^) of the whole aorta was removed and attached on Cell-Tak® coated glass, with the endothelial surface facing upwards. The artery preparation was bathed in PBS (PAA Clone). After preparation, the segments were stored in minimal essential medium supplemented with 20% FCS, 1% MEM vitamins, 1% MEM nonessential amino acids and 1% Pen-Strep.

### Immunostaining of heparan sulfates

As primary antibody, mouse anti-heparane sulfate (10E4 epitope) antibody (AMS Biotechnology, Abingdon Oxon, UK) and as secondary antibody, Qdot 655 goat anti-mouse antibody conjugate (Invitrogen), were used. GM 7373 cells were seeded on Ø35 mm cell culture glass bottom dishes (Willco, Amsterdam, Netherlands) for 72 h until a confluent cell monolayer was formed. The primary antibody (1∶500) was added to the living cells directly into the cell culture medium. After 30 min incubation at 37°C the cells were washed 3×5 min with PBS (37°C). Next, the secondary Qdot antibody conjugate was added (1∶800). The cells were again incubated for 30 min and washed 3×5 min in PBS at 37°C. PBS was removed and HEPES buffered solution (standard composition in mM: 135 NaCl; 5 KCl; 1 MgCl_2_; 1 CaCl_2_; 10 HEPES (N-2-hydroxyethylpiperazine-N′-2-ethanesulfonic acid); pH 7.4), supplemented with 1% FCS, was added.

### Simultaneous atomic force and immunofluorescence microscopy

**Figure 1:**
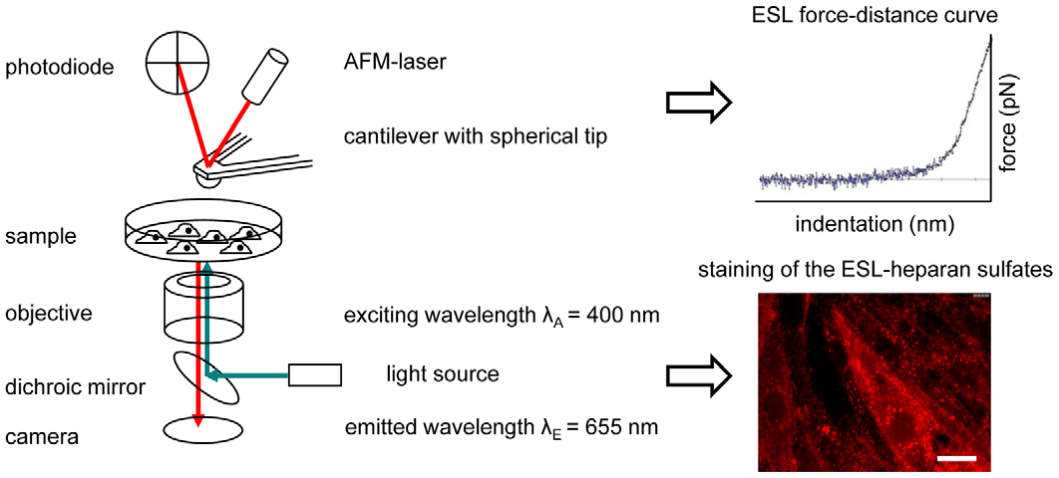
Combined atomic force and fluorescence microscopy. Using this setup, we performed force-distance measurements and immunofluorescence microscopy simultaneously in living endothelial cells to study the mechanical properties of the ESL (force-distance curve; upper right) in dependence of the amount of heparan sulfates (Qdot staining; lower right). The scale bar in the fluorescence image is 10 µm.

Using a BioScope Catalyst AFM (Bruker, Karlsruhe, Germany) combined with a Leica DMI 6000 B inverted fluorescence microscope (Leica Microsystems, Wetzlar, Germany), the nanomechanical properties of the ESL were monitored in dependence of the amount of heparan sulfates. Simultaneous indentation measurements and immunofluorescence microscopy (figure 1) were performed before and after the eGC of endothelial GM 7373 cells was enzymatically digested by heparinase I (1 SigmaU/ml = 0.002 U/ml, 45 min at 27°C). For AFM-measurements a soft cantilever (spring constant = 18 pN/nm; Novascan, Ames, IA, USA) with a spherical tip (sphere diameter = 1 µm) and a maximal loading force in the range of 400 pN was used. Qdot fluorescence was excited using a wavelength of 400 nm, whereas the emission was detected at 655 nm.

**Figure 2:**
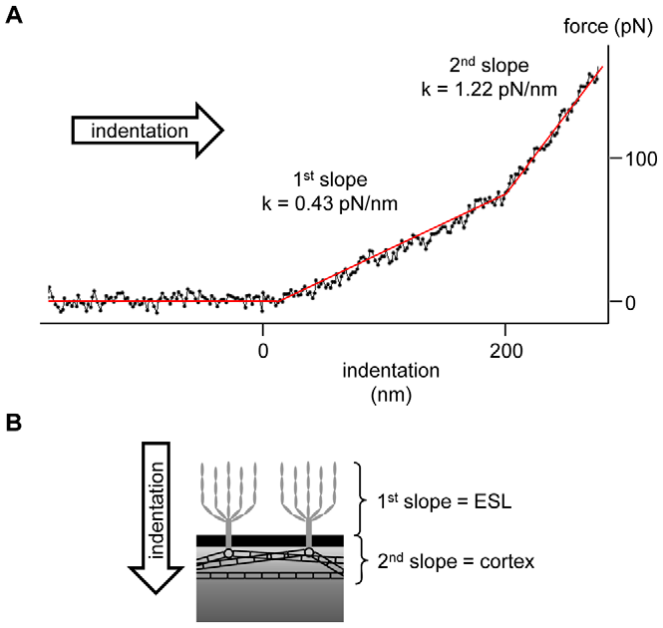
Schematic illustrating the different layers detectable by the indentation measurements. (A). Representative example of a force-distance curve obtained in a human endothelial cell (EA.hy 926 cell line) giving information about stiffness at defined indentation depths (B).

Indentation measurements provide information about *K*, the sample stiffness. *K* is the mechanical resistance of a sample against deformation along a defined degree of freedom:
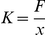
whereby *F* is the applied force and *x* is the displacement caused by the applied force to the sample. In this work, *K* is measured as the slope of the obtained force-distance curve (figure 2). Since cells consist of a vast number of different substructures and organelles, *K* depends strongly on indentation depth and location.

### AFM on *ex vivo* aortae

Force-distance measurements on murine *ex vivo* aortae were performed to examine the impact of the hawthorn extract WS 1442 on ESL stiffness and ESL height. For this series of experiments a Multimode AFM (Veeco, Mannheim, Germany) was used with the same parameters as for the Catalyst AFM setup. ESL stiffness and ESL height of murine *ex vivo* aortae were obtained before and after application of 10 µg/ml WS 1442 for 30 min.

### Sodium permeability assay

EA.hy 926 cells were grown on filter membranes (high density filter with 0.4 µm pore diameter; Greiner Bio One, Frickenhausen, Germany) in 12-well plates (Greiner Bio One, Frickenhausen, Germany) for 4 days. The control group was grown in standard cell culture medium, the WS 1442 group was grown in standard cell culture medium containing 10 µg/ml WS 1442. Prior to the experiment, medium with a sodium concentration of 155 mM (apical side) and 130 mM (basolateral side) was applied. Osmolarity was maintained constant by the addition of appropriate amounts of mannitol. After 2 hours of incubation at 37°C and 5% CO_2_, the basolateral sodium concentration was determined. The sodium concentration of the medium was obtained by means of a Radiometer EML 105 electrolyte analyser (Radiometer Copenhagen, Copenhagen, Denmark). The sodium permeability was calculated as follows:
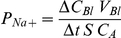
where Δ*C_BL_* is the change of the basolateral Na^+^ concentration and *C_A_* is the apical sodium concentration (considered to remain constant through the experiment). *V_BL_* is the basolateral fluid volume, *S* is the surface area of the filter membrane and Δ*t* the time of incubation. To test whether *P_Na+_* depends on the ESL, we applied heparinase I (1 SigmaU/ml, 37°C; Sigma-Aldrich Chemie GmbH, Munich, Germany) directly into the medium for 30 min prior to the experiment. Again, the medium was replaced with high sodium medium and *P_Na+_* was obtained as described above.

## Results

### Quantification of the nanomechanical properties of the ESL by AFM

**Figure 3:**
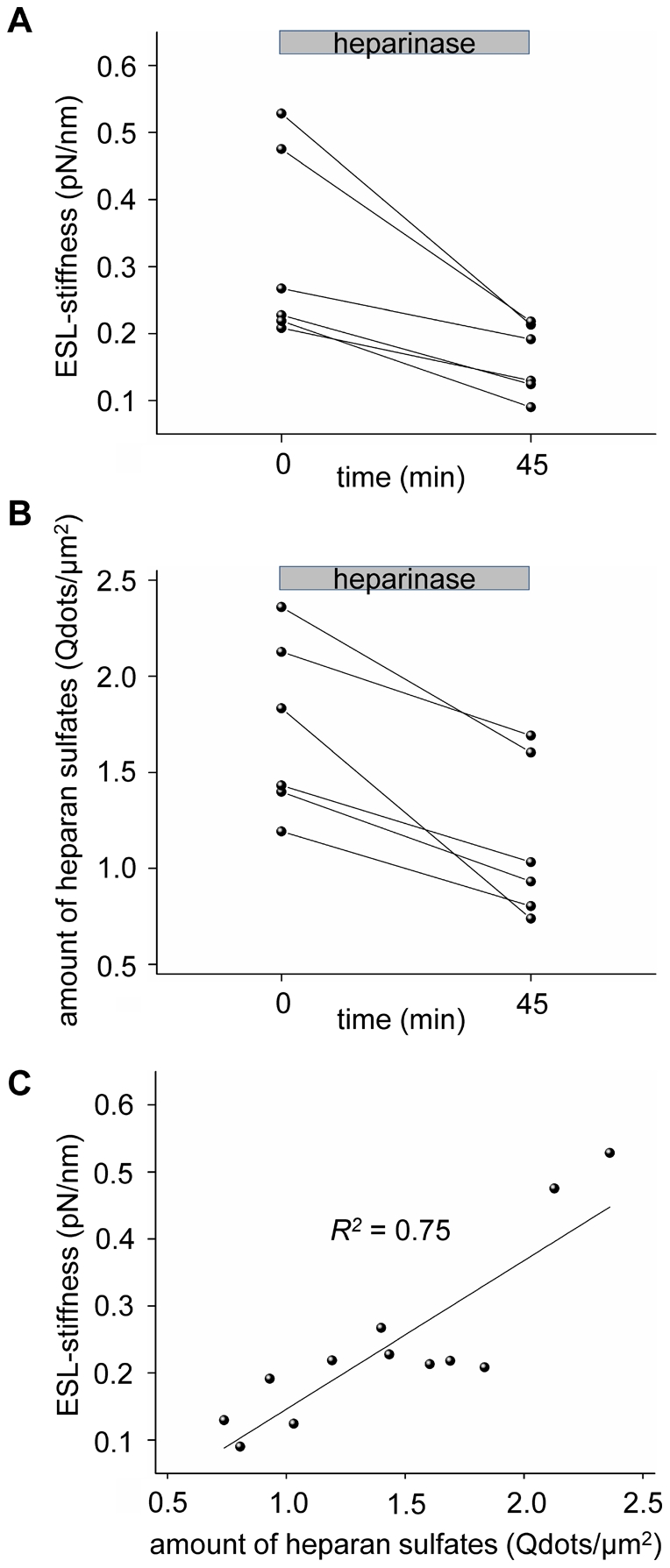
Effect of heparinase treatment on the ESL (n = 6, 10 measurements per n). After heparinase application for 45 min the ESL-stiffness (A) and the amount of heparan sulfate molecules (B) of the ESL decrease significantly. The amount of heparan sulfates strongly correlates with the ESL-stiffness (C; Spearman's correlation coefficient *R^2^* = 0.75, *p*<0.05).

The extracellular matrix of human cervical epithelial cells can be characterised by using AFM methods [36]. Our main goal was to establish a method to analyse the mechanical properties of the ESL by means of simultaneous AFM and immunofluorescence measurements. The data reveal a soft layer on the surface of the GM 7373 cells with a stiffness of about 0.32 pN/nm which is about 4 times softer than the cortical stiffness of endothelial cells. Heparinase treatment leads to a reduction in this stiffness by 47% (Figure 3A) as well as to a reduction in the heparan sulfate abundance on the cell surface by 34% (Figure 3B). This approach provides evidence that the ESL can be detected by AFM because the measured ESL stiffness and the amount of stained heparan sulfate decreased in an interdependent way after heparinase treatment. Furthermore, the ESL stiffness correlates strongly with the amount of stained heparan sulfates (Spearman's correlation coefficient *R^2^* = 0.75, *p*<0.01; Figure 3C). Similar experiments using AFM for measuring ESL stiffness have recently been published [15], [37].

### WS 1442 modulates the functional properties of the ESL

**Figure 4:**
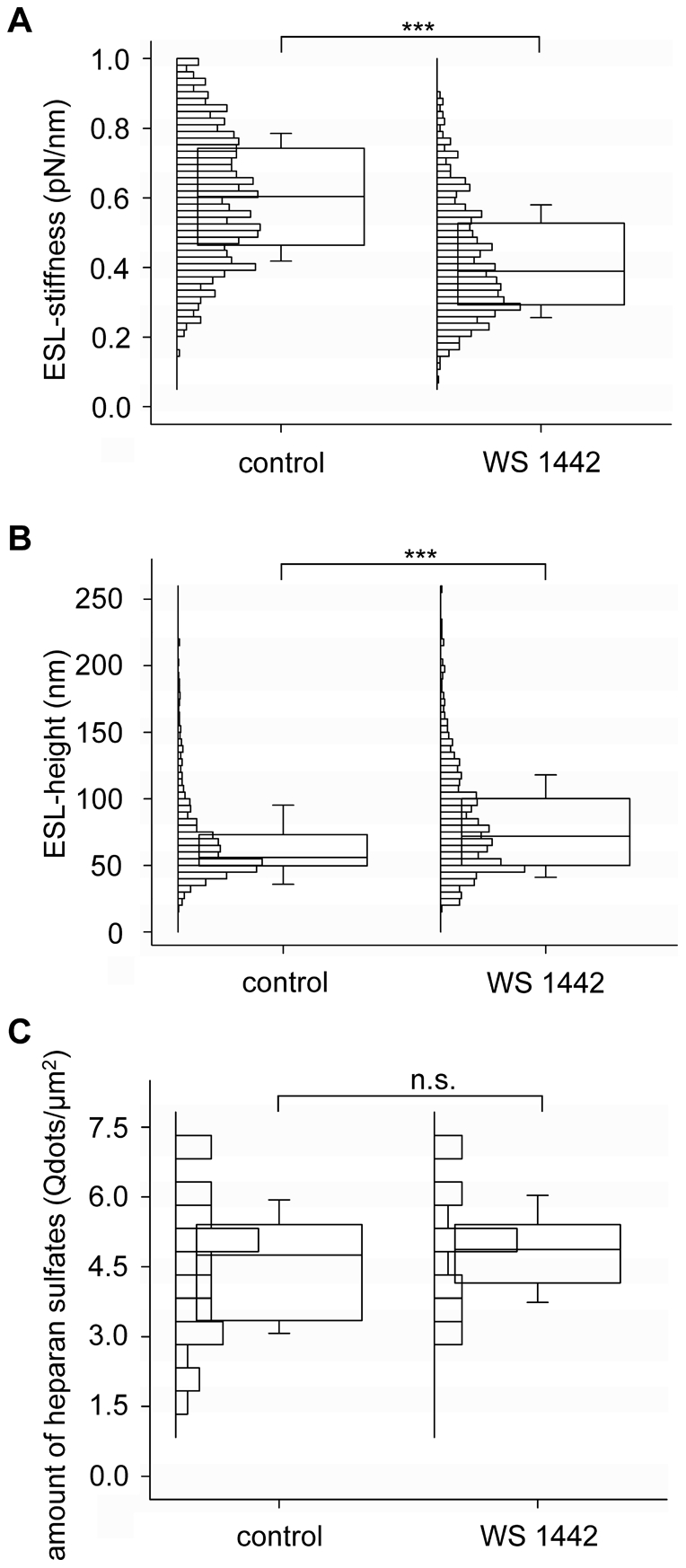
Impact of WS 1442 on the ESL-stiffness, the ESL-height (n = 36, 50 force-distance curves per n respectively) and the amount of heparan sulfates (n = 12). After administration of 10 µg/ml WS 1442 for 30 min the ESL becomes softer (A) and thicker (B), whereas the amount of heparan sulfates remains constant (C). Shown were median, IQR (box) and SD (whiskers).

Previous work has shown that WS 1442 exhibits vasoprotective properties. Some of these effects, such as an increase of NO-synthesis [17], [18] and cardioprotective effects in ischemic reperfusion injuries [20] could be related to the proper functioning of the ESL. To prove, whether WS 1442 has an impact on the ESL, we measured the mechanical properties of the endothelial surface layer by means of AFM. The advantage of the AFM is the possibility to measure living cells under physiological conditions. For the AFM experiments murine *ex vivo* arteries were used, in order to emulate physiological conditions as closely as possible. That it is feasible to use ex vivo arteries for AFM measurements has been shown previously [38]. Time-response experiments show that WS 1442 develops its maximal effect 30 min after administration (data not shown). The nanomechanical properties of the ESL were measured before and after administration of WS 1442 (10 µg/ml, 30 min, 37°C). For each group we measured six different sites on six different aortic preparations respectively, and each measurement represents an average of 50 force-distance curves. Measurements of the control group revealed a soft surface layer with an average stiffness of 0.60±0.007 pN/nm (mean ± SEM) and an average thickness of 65.54±1.06 nm. After WS 1442 treatment, the ESL becomes softer and thicker - the stiffness decreases by 30% to 0.42±0.006 pN/nm (Figure 4A) and the thickness rises by 22% to 79.54±1.32 nm (Figure 4B). These changes are highly significant (*p*<0.01, Mann-Whitney U-Test). Due to the lack of transparency of the aortic preparations, the simultaneous AFM and fluorescence microscopy could not be applied. To test, whether the changes of the mechanical properties of the ESL were due to conformational (e.g. collapse) or structural (e.g. loss of ESL constituents) alterations, we compared the amount of heparan sulfates between WS 1442-treated cells and untreated cells. Interestingly, the amount of heparan sulfates was not altered after 30 min of WS 1442 - treatment (Figure 4C).

### WS 1442 improves endothelial barrier function

**Figure 5:**
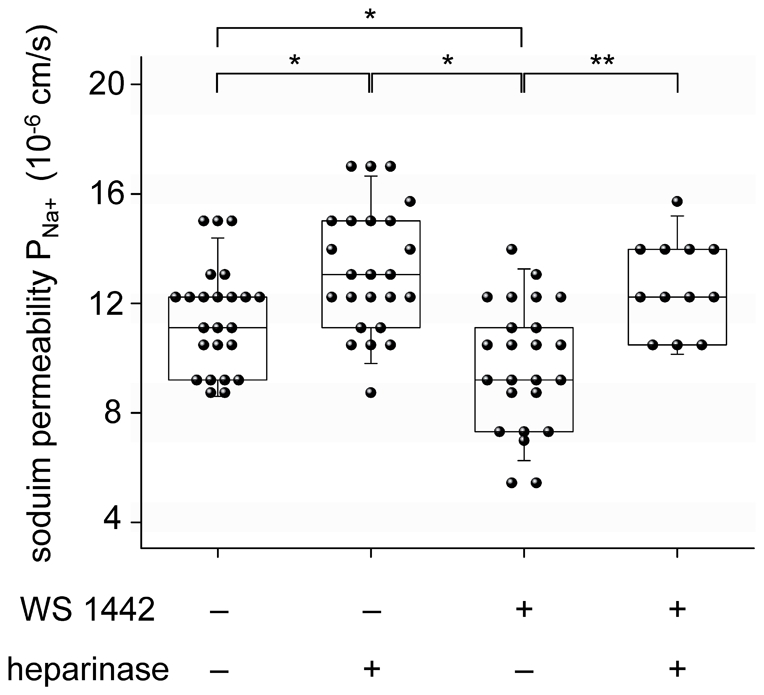
The sodium permeability *P_Na+_* of a cell monolayer (EA.hy 926 cells. Heparinase induced digestion of the ESL leads to an increase of *P_Na+_* (n = 24). In contrast, cells treated with 10 µg/ml WS 1442 show a lower *P_Na+_* (n = 24). The difference in *P_Na+_* between WS 1442-treated and untreated cells vanishes completely after enzymatic digestion of the ESL.

In a permeability assay we studied the *P_Na+_* of the endothelial cells before and after heparinase digestion of the ESL. The measured values (median and interquartile range (IQR)) are as follows: *P_Na+_* = 11.7*10^−6^ (IQR: 2.7*10^−6^, n = 24) cm/s for the control group and *P_Na+_* = 9.8*10^−6^ (IQR: 4.3*10^−6^, n = 24) cm/s for the WS 1442-treated group. After heparinase treatment, the *P_Na+_* of the control group rises by 12% to 13.1*10^−6^ (IQR: 3.6*10^−6^, n = 24) cm/s while the *P_Na+_* of the WS 1442-treated group rises by 24% to 12.2*10^−6^ (IQR: 3.1*10^−6^, n = 12) cm/s (Figure 5). Statistical comparison of the groups reveals the following: the ESL contributes to the *P_Na+_* of the endothelial cells because after enzymatically digestion of the eGC, *P_Na+_* is found to be significantly increased (*p*<0.017, Mann Whitney U-Test, Bonferroni corrected). WS 1442-treated endothelial cells show significantly lower *P_Na+_* (*p*<0.017) in contrast to the cells of the control group. This WS 1442 induced reduction of the *P_Na+_* is most likely caused by modifications of the ESL since both the control group and the WS 1442-treated group show no difference in *P_Na+_* after heparinase treatment.

## Discussion

### Influence of WS 1442 on the ESL

The results show that WS 1442 induces a change of the mechanical properties of the ESL within a few minutes. The ESL becomes 30% softer and 22% thicker. As WS 1442 does not influence the amount of heparan sulfate molecules at the surface of endothelial cells, it is assumed that the nature of the eGC-modulation is a result of conformational alterations. From our results it appears likely that the packing density of glycosaminoglycans in the ESL is a regulated process. Loss of ESL constituents like heparan sulfate leads to structural changes of the ESL and thus to decreased height and stiffness. In contrast, as observed after WS 1442 treatment, the amount of ESL constituents can remain also constant while height and stiffness change. A similar behaviour was observed by Sui et al. [39] for artificial surface-tethered polymer brushes. After solvent exchange the polymer brushes switch from a swollen to collapsed state within few seconds, leading to a decrease in thickness and an increase in stiffness of the polymer layer. So it is possible that the changes in the ESL mechanics measured in our study result from an alteration of the solvation state of the ESL. Changes in the mechanical properties of the ESL may lead to a change in haemodynamics [40]. A change in ESL could regulate processes on the cell surface such as receptor-ligand interactions. It is possible that the nanomechanical properties of the ESL influence the biochemistry of this structure, e.g. by regulating the number of binding sites for different blood-borne substances. The ESL also plays an important role in inflammatory processes [41], [42] which may explain the anti-inflammatory properties of WS 1442. Since the eGC is crucial for shear stress mechanotransduction [4]–[6], it is possible that a softer and thicker glycocalyx results in a better detection of shear stress by the endothelium and thus higher NO-synthesis and release.

With the use of *ex vivo* arteries physiological conditions are mimicked as closely as possible [38]. Nevertheless the *in vivo* situation may be different because of the absence of factors such as fluid shear stress or hormones *ex vivo*. Application of transmission electron microscopy shows that the ESL-height could be up to a few µm [43], in contrast to 40–300 nm measured in the present study. A possible explanation for this apparent discrepancy may be that the ESL itself is a multilayer structure containing layers of different composition and functional properties [44]. Our AFM approach, therefore, can detect stiffness only in those more dense ESL-regions which are close to the plasma membrane.

### Sodium permeability

As the results revealed that WS 1442 modulates the mechanical properties of the ESL, there was a need to test whether these modulations cause an alteration of the ESL-dependent endothelial barrier function. Comparison of the *P_Na+_* of control cells to those after heparinase digestion of the eGC does reveal that the ESL significantly contributes to the *P_Na+_* of the endothelium. In addition, the permeability depends on the physiological state of the ESL. This conclusion is derived from the observation that WS 1442 treated cells show a significantly lower *P_Na+_* in comparison to untreated cells. This difference completely disappeared after heparinase treatment, so it is assumed that the changes of *P_Na+_* are a result of modulation of the ESL. This conclusion is strengthened by the assumption that the total resistance *R_Na+_* - the reciprocal value of permeability (*R_Na+_ = 1/P_Na+_) -* of the endothelial cell monolayer is determined by the combined resistance of the ESL, of the endothelial cell layer itself and of the filter membrane of the inserts:
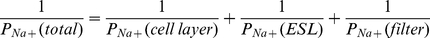



Because the sodium permeability of the filter membrane is extremely high, the term 1/*P_Na+_(filter)* is very small and thus negligible for further calculations. If it is assumed that after the ESL digestion *P_Na+_* depends only on the permeability of the endothelial cell itself, *P_Na+_(ESL)* can be calculated as follows:
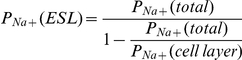

Table 1 summarizes the values for the measured *P_Na+_(total)*, *P_Na+_(cell layer)* and the calculated *P_Na+_(ESL)*. *In vivo*, a lower endothelial *P_Na+_* would result in a lower rate of sodium transfer from the blood across the endothelium to the interstitium. This mechanism may help retain sodium in the circulation and thus accelerate renal sodium excretion.

**Table 1:** Sodium permeability *P_Na+_* of human vascular endothelium (EA.hy 926 cell monolayer).

Group	*P_Na+_(total)* (10^−6^ cm/s) measured (IQR; n)	*P_Na+_(cell layer)* (10^−6^ cm/s) measured (IQR; n)	*P_Na+_(ESL)* (10^−6^ cm/s) calculated (IQR)
Control	11.7 (2.7; 24)	13.1 (3.6; 24)	110.4 (100.4)
WS 1442	9.8 (4.3; 24)	12.2 (3.1; 12)	50.4 (48.3)

Statistics (Mann Whitney U-Test with Bonferonni correction): *P_Na+_(total)* is significantly smaller than *P_Na+_(cell layer)* of control group (*p*<0.05) and WS 1442 group (*p*<0.01). *P_Na+_(total)* of the WS 1442 group is significantly smaller in comparison to *P_Na+_(total)* of the control group(*p*<0.05). There is no significant difference in *P_Na+_(cell layer)* between control group and WS 1442 group.

A bath sodium concentration in the high physiological range impairs endothelial cell function e.g. NO-synthesis [3]. In contrast to the effect of an increase in plasma sodium, WS 1442 increases endothelial NO-synthase activity [17], [18]. At the same time *P_Na+_* of the ESL decreases as shown in the present study. This may explain mechanistically how hawthorn extracts exert their protective effects on the cardiovascular system.

Vascular effects of WS 1442 have been related to its high content of oligomeric procyanidins (OPCs) (16). Red wine and purple grape juice polyphenols also exert beneficial effects on the cardiovascular system by enhancing NO-release [45] and inhibiting smooth muscle cell migration [46]. It is possible therefore that WS 1442 induced ESL modulation is mediated by OPCs. The identification of ESL modulating components will be necessary for further investigation of this issue.

**Figure 6:**
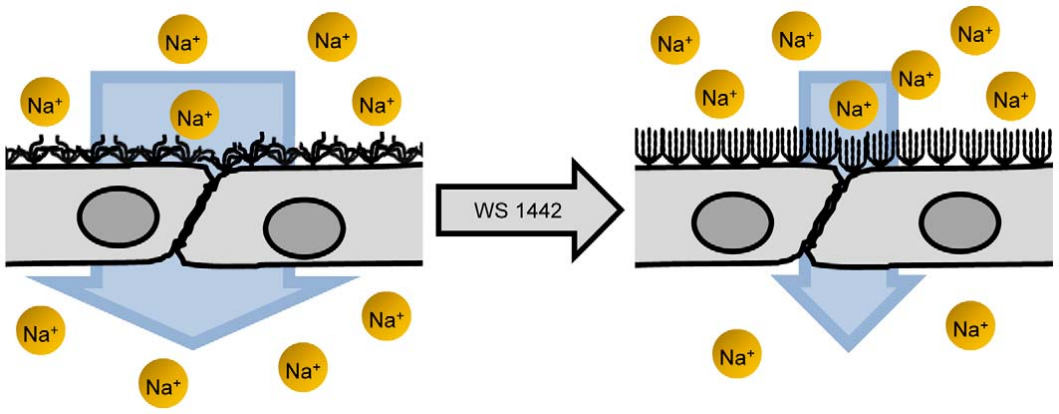
Model explaining the effect of WS 1442 on the conformation of the endothelial surface layer. The ESL can exist in a more densely packed state or in a more loosely packed state. The functional parameters of the ESL, such as e.g. the sodium permeability, strongly depend on the state of the ESL. WS 1442 induces a transition from the densely packed to the loosely packed state.

In conclusion, the results show that hawthorn extract WS 1442 leads to a softer and higher ESL companied by a reduction of the *P_Na+_(ESL)* (figure 6). This may explain some of the vasoprotective properties of the herbal extract.
