# Correction: Nanomechanics and Sodium Permeability of Endothelial Surface Layer Modulated by Hawthorn Extract WS 1442

**DOI:** 10.1371/annotation/ac261a1e-0beb-496a-b7ca-b099309776c8

**Published:** 2013-07-08

**Authors:** Wladimir Peters, Verena Drüppel, Kristina Kusche-Vihrog, Carola Schubert, Hans Oberleithner

The name of the second author was spelled incorrectly. The correct name is: Verena Drüppel. The correct citation is: Peters W, Drüppel V, Kusche-Vihrog K, Schubert C, Oberleithner H (2012) Nanomechanics and Sodium Permeability of Endothelial Surface Layer Modulated by Hawthorn Extract WS 1442. PLoS ONE 7(1): e29972. doi:10.1371/journal.pone.0029972.

